# Identification and evolutionary analysis of NAC transcription factors in *Eriobotrya japonica*: implications for sugar-acid regulatory networks during fruit development

**DOI:** 10.3389/fpls.2025.1671017

**Published:** 2025-09-26

**Authors:** Huiling Zhang, Meng Yang, Chengyang Ye, Minghui Chen, Hailing Gu, Xiurun Fan, Chencong Yang, Junwei Chen, Kai Xu, Boping Wu

**Affiliations:** ^1^ National Key Laboratory for Development and Utilization of Forest Food Resources, Collaborative Innovation Center for Efficient and Green Production of Agriculture in Mountainous Areas of Zhejiang Province, College of Horticulture Science, Zhejiang A&F University, Hangzhou, Zhejiang, China; ^2^ Institute of Horticulture, Zhejiang Academy of Agricultural Sciences, Hangzhou, Zhejiang, China

**Keywords:** NAC transcription factors, loquat, sugar-acid metabolism, fruit development, gene family

## Abstract

Loquat (*Eriobotrya japonica*) is an important subtropical evergreen fruit tree of the Rosaceae family that possesses significant edible and economic value. The NAC transcription factor family, as plant-specific regulatory factors, not only participated in plant growth and development but also played crucial roles in fruit quality formation. Through genome-wide analysis, this study identified 117 *NAC* family members in loquat, which were phylogenetically classified into 14 distinct subfamilies. Chromosomal localization revealed that 114 genes were unevenly distributed across 17 chromosomes, while the remaining 3 were located in genomic scaffold regions. Collinearity analysis indicated that loquat *NAC* genes primarily underwent purifying selection and showed high homology with *NAC* genes from other Rosaceae species. Cis-acting element prediction analysis suggested these genes were extensively involved in various biological processes, including abiotic stress responses, hormone signal transduction, and growth regulation. Expression pattern analysis based on transcriptome data further uncovered differential expression characteristics of *NAC* genes across different loquat cultivars and fruit developmental stages. Notably, correlation analysis identified several *NAC* candidate genes that were significantly associated with fruit sugar-acid content. This study provided the first comprehensive and systematic characterization of the *NAC* gene family in loquat, establishing an important foundation for elucidating the molecular mechanisms by which NAC transcription factors regulate loquat fruit flavor quality.

## Highlights

- First genome-wide identification and characterization of NAC transcription factors in loquat (*Eriobotrya japonica*).
- Comprehensive evolutionary analysis reveals conserved and divergent patterns in loquat *NAC* gene family.
- Expression profiling identifies candidate *NAC* genes associated with fruit sugar-acid metabolism.

## 1 Introduction

The NAC transcription factor family represented one of the largest and most characteristic families of transcriptional regulators in plants, named after its three initially identified members: NAM (No Apical Meristem), ATAF1/2 (Arabidopsis Transcription Activation Factor 1/2), and CUC2 (Cup-Shaped Cotyledon 2) (Xiong et al., 2025). These transcription factors possessed unique molecular structural features, comprising a highly conserved N-terminal DNA-binding domain and a highly variable C-terminal transcriptional regulation domain (Dorjee et al., 2024). This distinctive structural characteristic enabled NAC transcription factors to play pivotal regulatory roles in plant growth, development, and environmental adaptation.

Numerous studies demonstrated that NAC transcription factors participated in the regulation of multiple critical physiological processes in plants through complex regulatory networks, including but not limited to: seed dormancy and germination, flowering time determination, organ morphogenesis, and fruit development (Zhang et al., 2025). Particularly in Rosaceae fruit trees, NAC transcription factors were confirmed as key molecular switches regulating fruit quality formation. In apple (*Malus domestica*), MdNAC18.1 promoted fruit ripening by activating genes involved in ethylene biosynthesis pathways (Yue et al., 2025); in pear (*Pyrus pyrifolia*), PpNAC187 was shown to participate in regulating the lignification process during fruit cork spot development stage (Li et al., 2019); while in peach (*Prunus persica*), PpNAP6 influenced fruit ripening through ethylene synthesis regulation (Dai et al., 2023), and PpNAP4 directly activated promoters of carotenoid biosynthesis genes to participate in fruit coloration (Dai et al., 2025b). Notably, these regulatory functions often exhibited species specificity, such as MdNAC77L specifically promoting anthocyanin accumulation in apple and strawberry (Shi et al., 2024), while PavNAC56 in sweet cherry (*Prunus avium*) specifically regulated fruit softening (Qi et al., 2022).

Among various factors determining fruit quality, sugar-acid metabolic balance was one of the most crucial determinants. Recent studies revealed the central role of NAC transcription factors in regulating sugar-acid metabolism. In strawberry (*Fragaria* × *ananassa*), FvNAC073 affected sucrose accumulation by differentially regulating *FvSPS1* and *FvSUS2* expression (Xiao et al., 2025); in watermelon (*Citrullus lanatus*), knockout of ClNAC68 significantly reduced soluble solid content and sucrose accumulation in fruit flesh (Wang et al., 2021); while in apple, MdNAC5 promoted sucrose hydrolysis by activating *MdNINV6* transcription (Zhang et al., 2024). Regarding organic acid metabolism, PpBL in peach synergistically activated *PpALMT4* expression with *PpNAC1* to promote malic acid accumulation (Chen et al., 2025); mutation of *AcNAC1* in kiwifruit (*Actinidia* spp.) led to a significant reduction in citric acid content (Fu et al., 2023); and the complex formed by CitNAC62 and CitWRKY1 in citrus affected citric acid degradation by regulating *CitAco3* expression (Li et al., 2017). Particularly noteworthy was that EjNAC25 in loquat simultaneously regulated two key processes-sugar metabolism (promoting sucrose hydrolysis) and acid metabolism (promoting malic acid accumulation)-by directly activating *EjNI* and *EjtDT2* expression (Chi et al., 2025), providing important clues for studying the role of NAC transcription factors in coordinated sugar-acid regulation.

Loquat (*Eriobotrya japonica*), as an economically important fruit species in the Maloideae subfamily of Rosaceae, has its fruit flavor quality primarily governed by the dynamic equilibrium of sugar-acid components. With consumers’ growing demands for fruit quality, elucidating the molecular regulatory mechanisms of sugar-acid metabolism in loquat has become a crucial frontier in pomological research. While the functions of NAC transcription factors have been extensively investigated in other Rosaceae fruit trees, several critical scientific questions remain unresolved regarding the systematic identification of the *NAC* gene family in loquat and its regulatory mechanisms in fruit quality formation, including the composition and evolutionary characteristics of the *NAC* gene family in loquat, the correlation between expression patterns of different *NAC* members during fruit development and sugar-acid accumulation, and the specific molecular mechanisms through which key NAC members regulate sugar-acid metabolism.

This study employed an integrated multi-omics approach combining comparative genomics, transcriptomics, and metabolomics to conduct the first genome-wide systematic identification and functional analysis of the *NAC* gene family in loquat. The research comprehensively identified *NAC* gene family members and analyzed their evolutionary characteristics based on the latest loquat genome data. It screened key NAC members involved in sugar-acid metabolism regulation through multi-cultivar, multi-developmental stage transcriptome and metabolome association analysis. These results not only enriched our understanding of the evolution and functional differentiation of transcription factors in Rosaceae plants but also provided important theoretical basis and genetic resources for molecular breeding of loquat flavor quality.

## 2 Materials and methods

### 2.1 Plant materials

Two loquat (*Eriobotrya japonica*) cultivars with distinct ripening characteristics were selected for this study: ‘YingShuang’ (‘YS’), an early-maturing white-fleshed variety (harvested around May 10^th^), and ‘ZheHong No.16’ (‘ZH’), a mid-season red-fleshed variety (harvested around May 25^th^). The differences in maturity period and flesh traits provide an ideal system for investigating the expression patterns of genes at different fruit developmental stages, as well as their regulatory roles in sugar-acid metabolism. Furthermore, as representative cultivated varieties of loquat, their well-documented agronomic backgrounds enhance the biological relevance and potential applicability of our research findings. Fruits were collected at four developmental stages according to (Liu et al., 2024a). For each stage, three biological replicates (five fruits per replicate) were immediately frozen in liquid nitrogen and stored at -80°C for subsequent analysis.

### 2.2 Identification of loquat *NAC* genes

The genome data of loquat were obtained from the GigaScience Database (http://gigadb.org/dataset/view/id/100711). The amino acid sequences of *Arabidopsis* NAC family members were obtained from the Arabidopsis Information Resource (TAIR, https://www.arabidopsis.org/). Hidden Markov models (HMMs) of the NAC domain (PF01489) and NAM domain (PF02365) were downloaded from PFAM (http://pfam.sanger.ac.uk/) for the identification of loquat *NAC* members. BLAST was further employed to screen for loquat *NAC* genes. Members containing at least one NAM domain were selected using the NCBI Conserved Domain Database (CDD, http://www.ncbi.nlm.nih.gov). The physicochemical properties of *NAC* genes, including molecular weight, isoelectric point, and amino acid length, were predicted online using the ExPASy server (https://web.expasy.org/protparam/). Subcellular localization predictions were conducted on the WoLFPSORT platform (https://wolfpsort.hgc.jp/).

### 2.3 Phylogenetic analysis and chromosomal localization of *NAC* genes

Multiple sequence alignment of NAC protein sequences from loquat and *Arabidopsis* was conducted using MEGA 11.0. Phylogenetic reconstruction was performed employing the neighbor-joining (NJ) method with 1,000 bootstrap replicates for node support estimation. The resultant phylogenetic tree was subsequently visualized and annotated using the Interactive Tree of Life (iTOL) web platform (https://itol.embl.de/). Chromosomal localization of the identified loquat NAC members was performed using TBtools software, with reference to the chromosomal position information provided in the loquat genome annotation file.

### 2.4 Gene structure and conserved motif analysis of *NAC* genes

Gene structure analysis of loquat NAC family members was performed using TBtools, with visualization of exon-intron organization. Conserved protein motifs were subsequently identified through MEME Suite (v5.5.2, https://meme-suite.org/) with the following parameters: maximum number of motifs set to 10, motif width ranging from 6 to 50 amino acids, and E-value threshold < 1e-10.

### 2.5 Collinear and *cis*-acting regulatory elements analysis of *NAC* genes

Intraspecies synteny analysis of loquat *NAC* genes was performed using the One-Step MCScanX-SuperFast algorithm implemented in TBtools (v2.018). For cross-species comparison, intergenomic syntenic relationships were investigated through Advanced Circos visualization in the same platform. Promoter regions (2,000 bp upstream of transcription start sites) were extracted using TBtools and subsequently analyzed for cis-regulatory elements via PlantCARE (https://bioinformatics.psb.ugent.be/webtools/plantcare/html/). Final visualization of all predicted regulatory elements was conducted using TBtools’ integrated graphics modules.

### 2.6 Transcriptomic profiling of *NAC* gene expression patterns in loquat

To elucidate the expression dynamics of *NAC* genes in loquat, we performed RNA-seq analysis on fruit samples from different cultivars at key developmental stages. Total RNA was extracted using the CTAB method and subjected to quality control (RIN≥7.0, OD260/280 = 1.8-2.0, RNA amount≥1μg). mRNA was enriched using oligo (dT) beads and fragmented, followed by double-stranded cDNA synthesis with M-MuLV Reverse Transcriptase and DNA Polymerase I. After end repair, A-tailing, and adapter ligation, the libraries were quality-checked using Qubit and Bioanalyzer before 150bp paired-end sequencing on the NovaSeq 6000 platform. Raw data were processed through quality control (fastp v0.20.0), genome alignment (HISAT2 v2.0.5), and FPKM quantification (StringTie v1.3.3b). Differential expression analysis was conducted using DESeq2 (v1.20.0) with thresholds of adjusted *p*-value<0.05 and |log2FoldChange|>1.

### 2.7 Determination of sugar and acid content

For sugar and acid content analysis, samples (0.1 g) were first extracted in 1.4 mL of methanol at 70°C for 15 min and then centrifuged at 11,000 × g for 10 min. The resulting supernatant was phase-separated by adding 1.5 mL of H_2_O and 750 μL of chloroform, followed by centrifugation at 2,200 × g for 10 min. The aqueous phase (1 mL) was collected and stored at -80°C prior to analysis. For derivatization, 100 μL of the extract was combined with 10 μL of ribitol (0.2 mg/mL) as an internal standard, dried under nitrogen, and then sequentially derivatized by first reacting with 60 μL of methoxyamine hydrochloride (20 mg/mL in pyridine) at 37°C with shaking (950 rpm) for 1.5 h, followed by silylation with 40 μL of BSTFA containing 1% TMCS under the same conditions for 30 min. The derivatized samples were transferred to GC vials and analyzed via gas chromatography within 24 h to ensure sample stability.

### 2.8 Statistical analysis

Data visualization was performed using OriginPro 2023 and ChiPlot (https://www.chiplot.online/). Pearson correlation analysis was applied to assess the linear relationships between the expression levels of *NAC* genes and the content of sugar-acid components (including fructose, glucose, sucrose, malic acid, and citric acid). Correlations with a *p*-value < 0.05 were considered statistically significant. These significant associations are explicitly highlighted in the heatmaps, where the correlation coefficients (*R*-values) reflect both the strength and direction of the relationships.

## 3 Results

### 3.1 Identification of *NAC* genes in loquat

**Figure 1:**
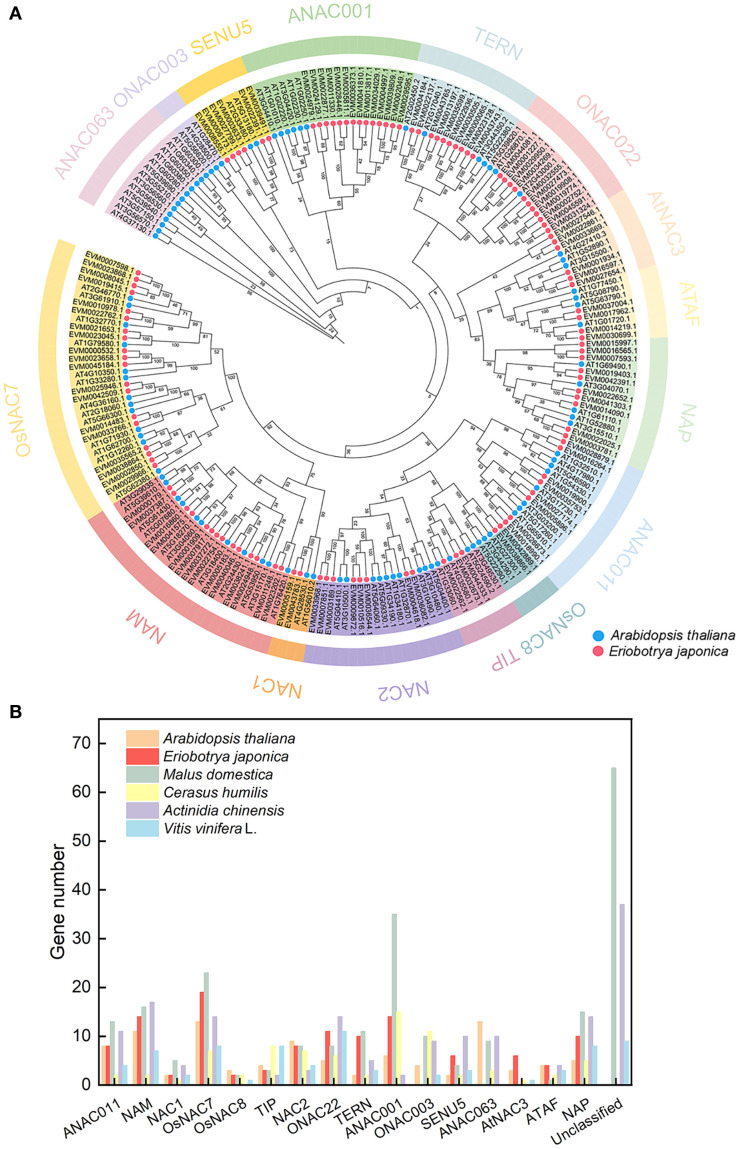
Phylogenetic analysis of the NAC family across multiple species. **(A)** Phylogenetic analysis of NAC proteins in loquat and *Arabidopsis thaliana*. Red circles represent loquat NAC proteins; blue circles represent *Arabidopsis* NAC proteins. **(B)** Distribution of plant *NAC* genes across phylogenetic groups.

Through comprehensive genome-wide screening, a total of 117 NAC transcription factor family members were identified in the loquat (*Eriobotrya japonica*) genome (**Figure 1**). Detailed characterization revealed substantial variation in the physicochemical properties of these proteins (**Supplementary Table 1**). Among them, protein length ranged from 201 amino acids (EVM0003633.1) to 851 amino acids (EVM0022562.1), representing the shortest and longest isoforms respectively. Molecular weights varied from 22.9 kDa (EVM0003633.1) to 94.4 kDa (EVM0022562.1), with an average of 42.9 kDa across all members. Theoretical isoelectric points (pI) spanned from 4.54 (acidic, EVM0039809.1) to 9.27 (basic, EVM0007593.1). The mean pI of 6.58 suggested a balanced distribution of acidic and basic NAC members. Subcellular localization prediction showed that the majority of identified NAC members (99 genes, 83.89%) exhibited nuclear localization, which aligns well with their canonical role as transcription regulators. This predominant nuclear targeting pattern strongly supported their predicted function in DNA binding and transcriptional regulation. The remaining NAC proteins showed distinct localization patterns to other cellular compartments (cytoplasm, chloroplasts, vacuoles, and endoplasmic reticulum), suggesting potential functional diversification beyond transcriptional control.

### 3.2 Phylogenetic analysis and Comparative genomics of *NAC* genes

To elucidate the evolutionary relationships of *NAC* genes in loquat, we conducted a phylogenetic analysis by aligning NAC protein sequences from loquat with *Arabidopsis thaliana*, followed by constructing a neighbor-joining tree (**Figure 1A**). The resulting phylogeny classified the 117 loquat NAC proteins into 14 distinct subfamilies, including ATAF, AtNAC3, ONAC022, TERN, ANAC001, SENU5, OsNAC7, NAM, NAC1, NAC2, TIP, OsNAC8, ANAC011, and NAP. Among these, the OsNAC7 subfamily was the largest, containing 19 members, while the NAC1 and OsNAC8 subfamilies were the smallest, each with only two genes.

Comparative analysis across multiple species revealed significant variation in *NAC* gene family size, ranging from 74 members in grape (*Vitis vinifera*) to 228 in apple (*Malus domestica*) (**Figure 1B**; **Supplementary Table 2**). The OsNAC7 subfamily, which was highly represented in loquat (19 members, accounting for 16.24%), was also prominent in *Arabidopsis*. However, the ONAC003, ANAC063, and Unclassified subfamily, present in other species, were entirely absent in loquat. Remarkably, comparative genomic analysis revealed a striking expansion of both NAC2 and ANAC001 subfamilies in Rosaceae fruit trees (*Malus domestica*: 8 NAC2, 35 ANAC001; *Cerasus humilis*: 7 NAC2, 15 ANAC001; *Eriobotrya japonica*: 8 NAC2, 14 ANAC001) compared to non-Rosaceae species (*Actinidia chinensis*: 3 NAC2, 2 ANAC001; *Vitis vinifera*: 4 NAC2, 0 ANAC001). This represented a 2-3-fold expansion of the NAC2 subfamily and an even more pronounced expansion (up to 17-fold) of the ANAC001 subfamily in Rosaceae species.

### 3.3 Chromosomal distribution and organization of *NAC* genes in loquat

**Figure 2:**
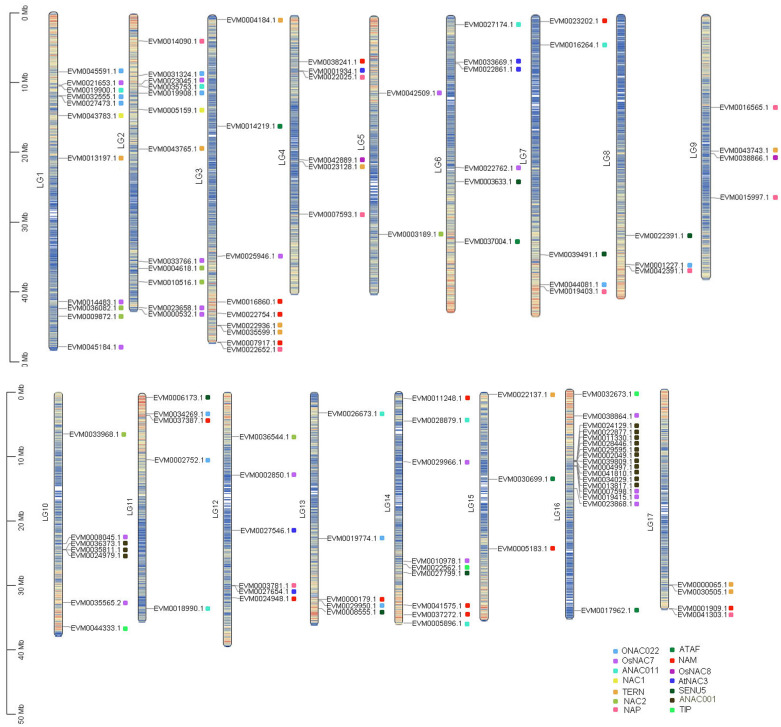
Chromosomal localization of *NAC* genes in loquat.

Genome-wide chromosomal mapping revealed an uneven distribution pattern of *NAC* genes across the loquat genome (**Figure 2**). Among the 117 identified *NAC* genes, 114 were successfully anchored to the 17 loquat chromosomes, while the remaining 3 genes were located on unassembled scaffold regions. The distribution exhibited remarkable chromosomal bias, with LG16 harboring the highest number of *NAC* genes (17 genes), followed by LG2 (12 genes) and LG1 (11 genes). In contrast, LG5 contained only two *NAC* genes, representing the most gene-poor chromosome for this family. Notably, the ANAC001 subfamily showed a striking localization pattern, with 11 of its members (64.8% of the subfamily) clustered on LG16 and the remaining 3 members exclusively located on LG10. This observation suggested potential subfamily-specific expansion events through localized gene duplication. Other chromosomes displayed intermediate *NAC* gene densities, including LG3 and LG14 (9 genes each), LG10 (7 genes), and LG4, LG6, and LG12 (4 genes each).

### 3.4 Collinear analysis of *NAC* genes in loquat

**Figure 3:**
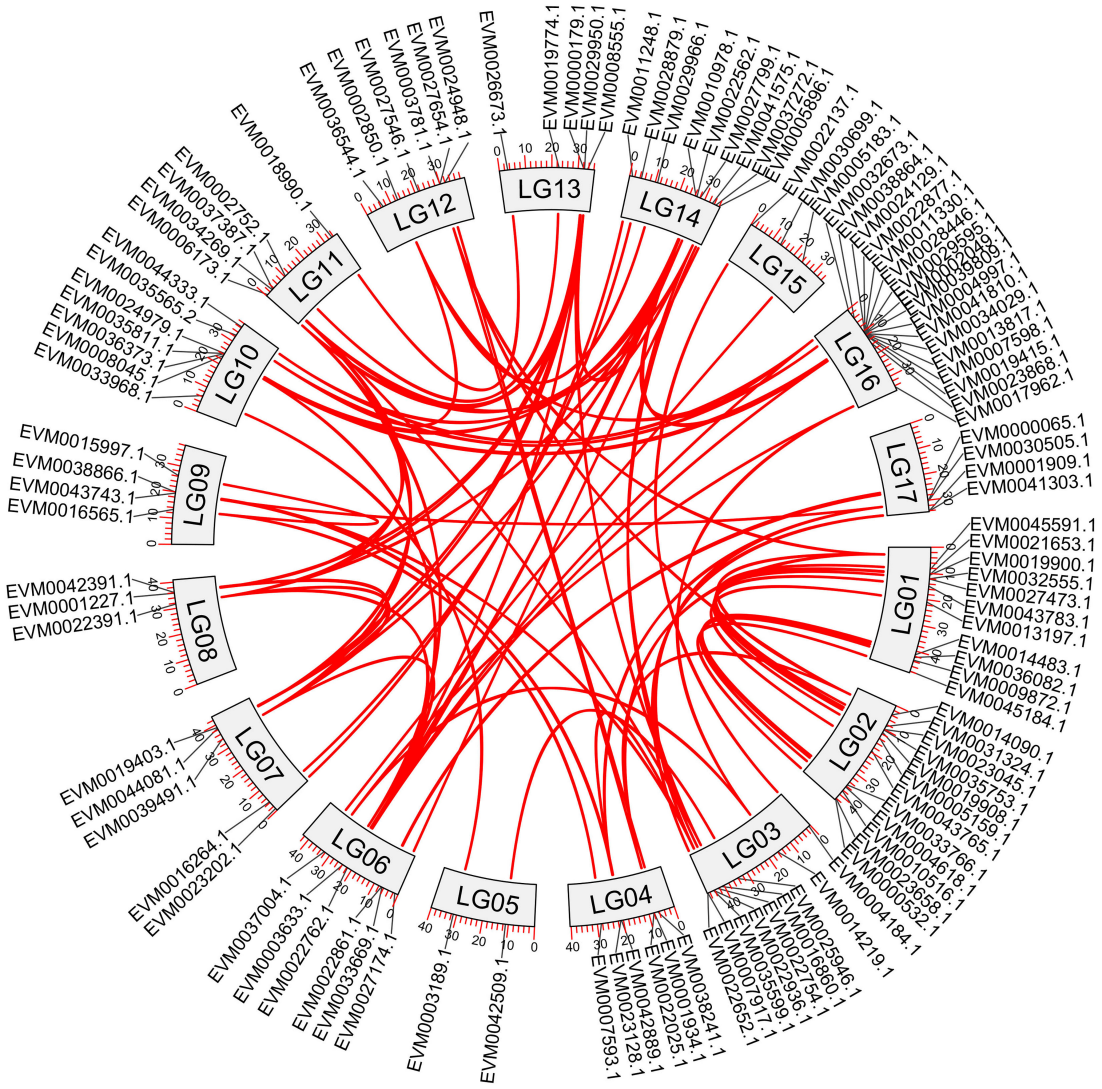
Intraspecific collinearity analysis of *NAC* genes in loquat. Red lines represent collinear relationships among *NAC* genes.

A total of 90 duplication events were identified within the loquat *NAC* genes, including both segmental and tandem duplications (**Figure 3**). Selective pressure analysis based on *K*a/*K*s ratios demonstrated that 113 out of the examined *NAC* genes exhibited *K*a/*K*s values < 1.0 (**Supplementary Table 3**), strongly suggesting the predominance of purifying selection in the evolution of these genes.

**Figure 4:**
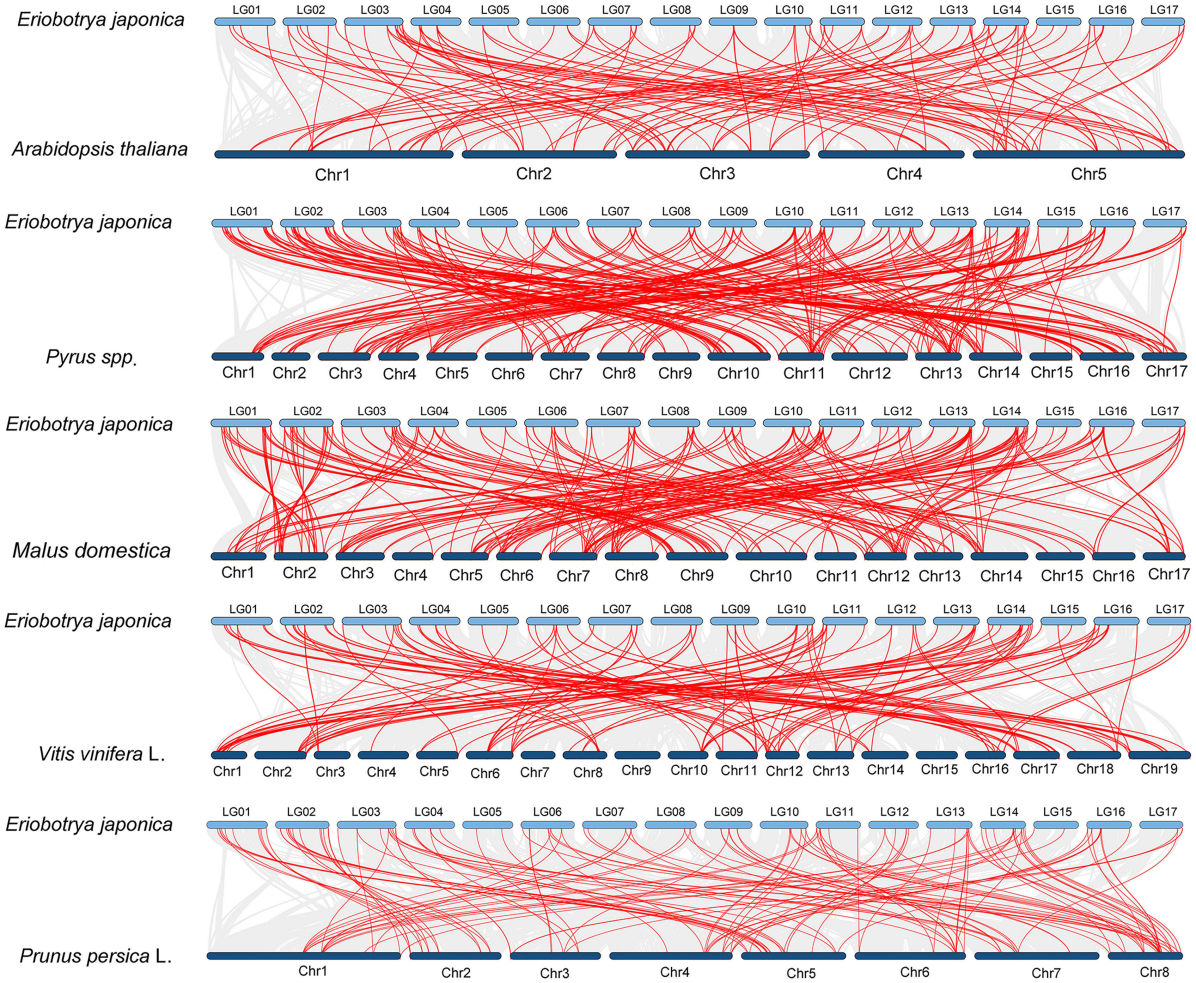
Comparative collinearity analysis of *NAC* genes between loquat and five representative species. Red lines depict collinear gene pairs.

Comparative collinearity analysis revealed differential conservation patterns of *NAC* genes among species (**Figure 4**). Of particular note, loquat showed the strongest synteny with pear (*Pyrus* spp.), sharing 248 homologous gene pairs, followed closely by apple (*Malus domestica*) with 231 pairs. This contrasted with the lower numbers observed for peach (*Prunus persica*; 133 pairs), grape (*Vitis vinifera*; 131 pairs), and *Arabidopsis thaliana* (116 pairs).

### 3.5 Structural characterization of *NAC* genes in loquat

**Figure 5:**
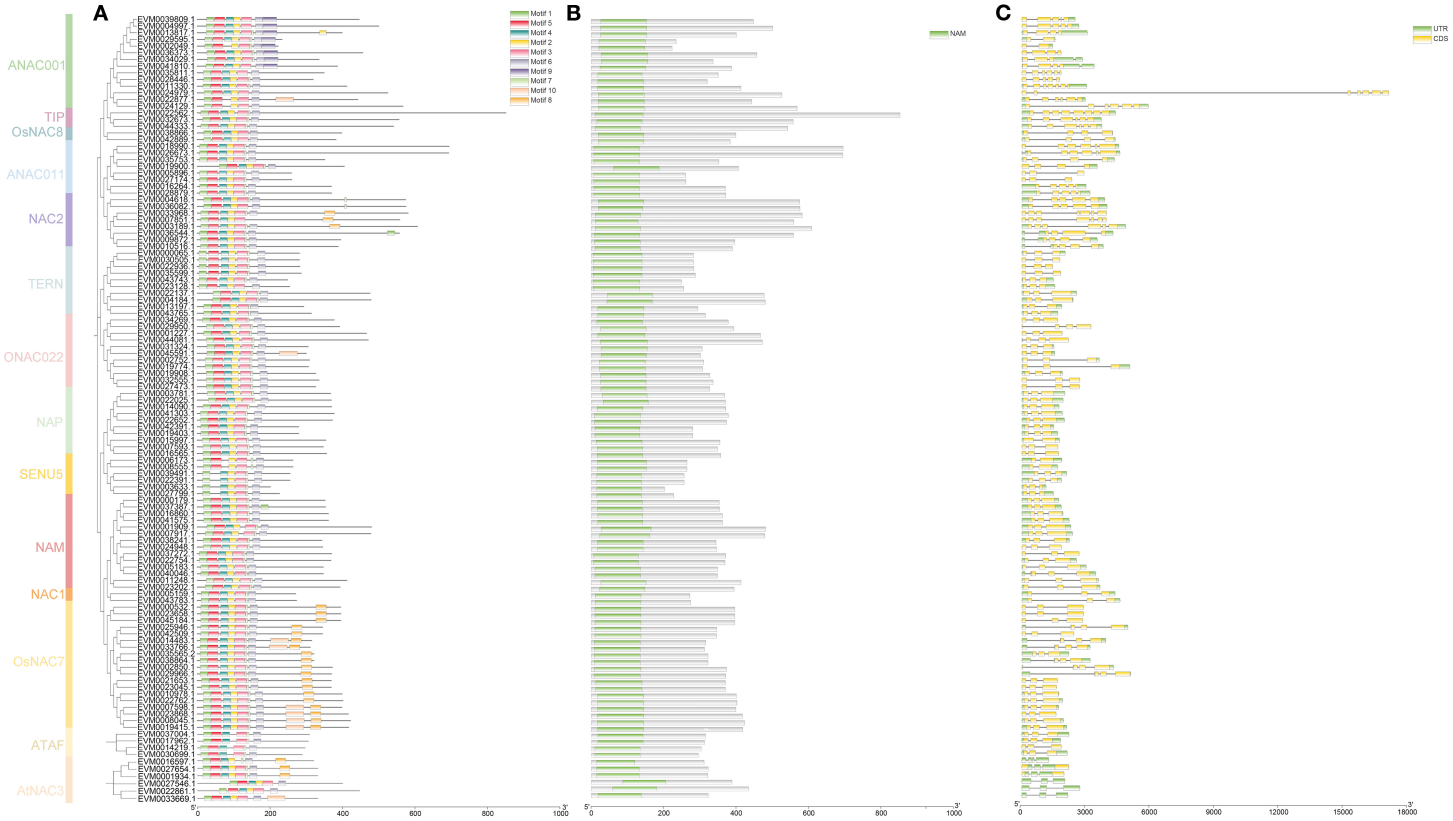
Motifs, domains, and gene structures of the *NAC* genes in loquat. **(A)** Distribution of conserved motifs in loquat NAC proteins. **(B)** Protein domain architectures of loquat *NAC* genes. **(C)** Exon-intron structures of loquat *NAC* genes.

Conserved motif analysis revealed distinct architectural features among *NAC* family members (**Figure 5A**). Notably, motifs 1 and 6 demonstrated universal conservation across all members, suggesting their fundamental functional significance. Motifs 3 (present in 116 members, accounting for 99.15%) and 2 (113 members, 96.58%) showed broad distribution patterns, while motifs 8 (25 members, 21.37%), 9 (8 members, 6.84%), and 10 (9 members, 7.69%) exhibited restricted occurrence, implying potential subgroup-specific functional specialization. Phylogenetic analysis further indicated that closely related subgroups maintained highly conserved motif architectures, supporting their functional coherence. All identified NAC proteins contained the characteristic NAM domain, confirming their classification within this transcription factor family (**Figure 5B**). Gene structure analysis uncovered remarkable diversity in exon-intron organization (**Figure 5C**). Four members (3.41%), exclusively from the AtNAC3 subgroup, were completely intronless. The remaining genes displayed varying exon numbers: 26 members (22.22%) were exonless, while others contained either one (8 members), two (61 members), or three exons (12 members). This structural heterogeneity provides valuable insights into the evolutionary diversification of loquat *NAC* genes.

### 3.6 *Cis*-acting regulatory element analysis of loquat *NAC* genes

**Figure 6:**
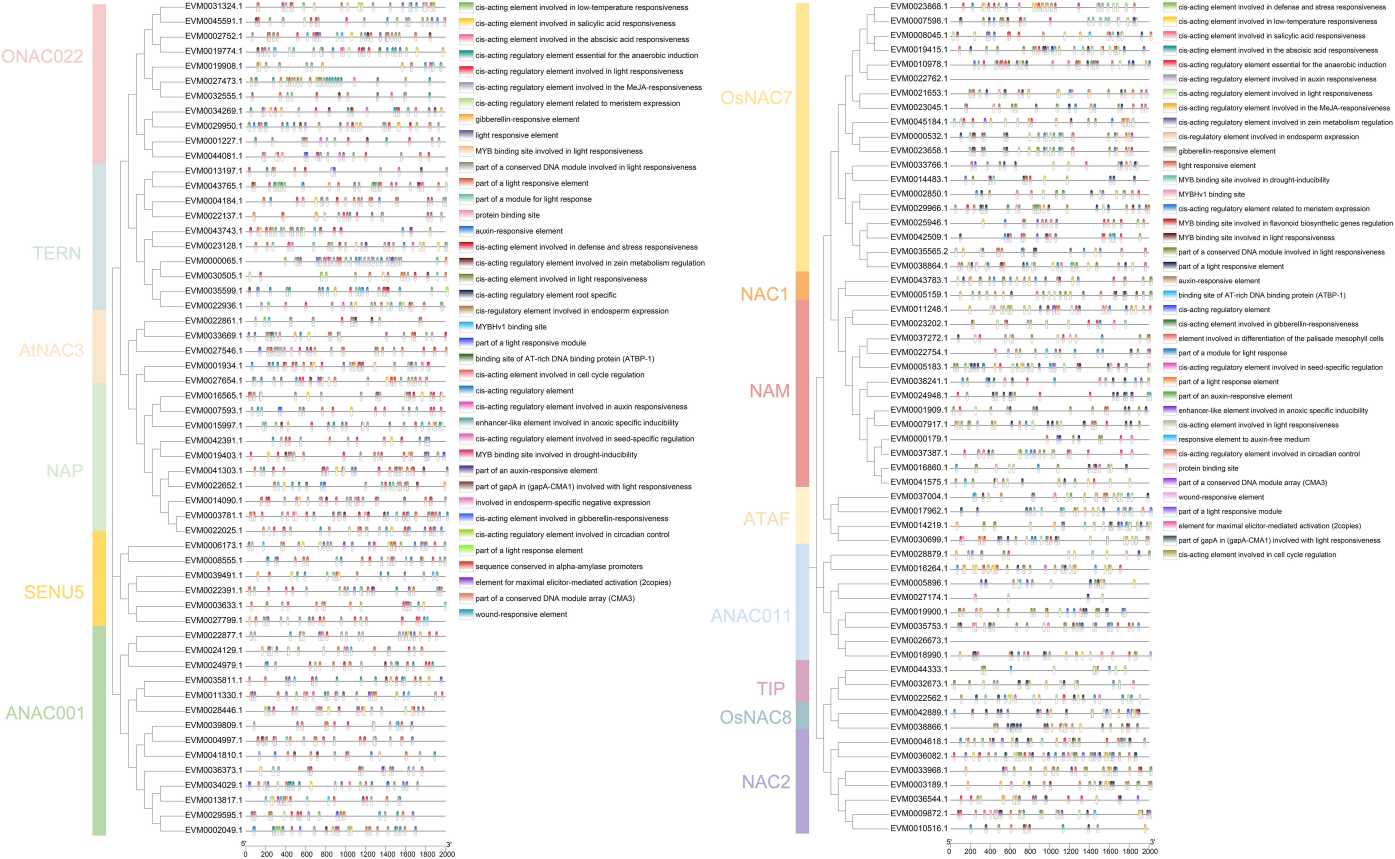
*Cis*-acting elements in the promoter regions of *NAC* genes in loquat. Boxes of different colors represent distinct types of cis-acting elements.

*Cis*-acting element analysis provided valuable insights into the potential functions of genes (**Figure 6**). The results demonstrated that among 114 genes (excluding three genes without promoter regions), a total of 3,292 *cis*-acting elements belonging to 40 categories were predicted. Notably, no *cis*-acting elements were detected in two members from the OsNAC7 (*EVM0022762.1*) and ANAC011 (*EVM0026673.1*) subgroups. These elements were predominantly associated with hormone responses, growth and development, as well as biotic and abiotic stress responses. Quantitative analysis revealed light-responsive elements as the most abundant category (1,456 elements, 44.2%), followed by methyl jasmonate-responsive (401 elements, 12.1%) and abscisic acid-responsive elements (375 elements, 11.4%). Additionally, auxin-responsive elements (96, 2.9%) and gibberellin-responsive elements (90, 2.7%) associated with fruit ripening were identified in the study. In the distribution analysis of regulatory elements, the highest number of gibberellin-responsive elements (4 in total) was predicted in the promoter region of gene *EVM0005159.1*, which is classified under the NAC1 subgroup. Conversely, the maximum count of auxin-responsive elements (3 per gene) was detected in multiple genes, including *EVM0007598.1* and *EVM0019415.1* from the OsNAC7 group, *EVM0043743.1* from the TERN subgroup, *EVM0033968.1* from the NAC2 subgroup, and *EVM0027654.1* from the AtNAC3 subgroup. These findings suggested that NAC transcription factors in loquat likely participate in the regulation of diverse physiological processes through these cis-regulatory mechanisms.

### 3.7 Expression pattern analysis of *NAC* genes in loquat fruits

**Figure 7:**
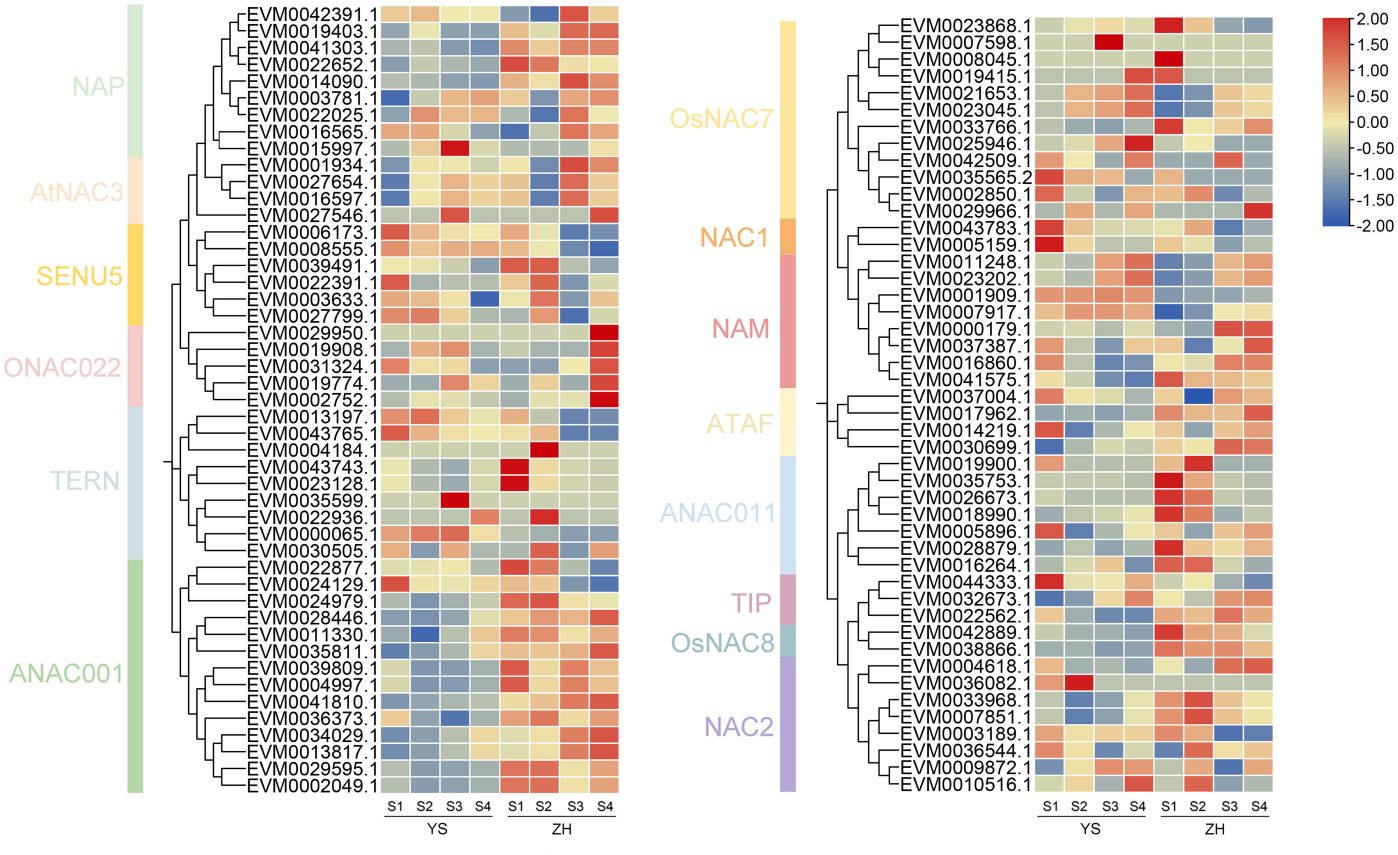
Expression patterns of *NAC* genes at different developmental stages in two cultivars. Red indicates high expression levels, while blue represents low expression levels.

Systematic analysis of the *NAC* gene family expression profiles in loquat revealed distinct cultivar-specific expression patterns and dynamic regulation during fruit development (**Figure 7**). Among the 117 identified *NAC* genes, 93 (79.5%) showed expression activity in fruit tissues, while 24 (20.5%) remained transcriptionally silent. It was worth noting that six genes exhibiting cultivar-specific expression patterns. Three genes were specifically expressed in ‘YS’, belonging to TERN (*EVM0035599.1*), OsNAC7 (*EVM0007598.1*), and NAC2 (*EVM0036082.1*) subfamilies, while three other genes were uniquely expressed in ‘ZH’, including members of ONAC022 (*EVM0029950.1*), TERN (*EVM0004184.1*), and OsNAC7 (*EVM0008045.1*) subfamilies. During fruit development, nine genes showed elevated expression during early developmental stages, including three SENU5 subfamily members, two TERN subfamily members, and single representatives from OsNAC7, NAC1, and ANAC011 subfamilies. In contrast, 11 genes exhibited significantly upregulated expression during late maturation stages, predominantly from ANAC001 (4 members), OsNAC7 (2 members), and NAM (2 members) subfamilies, and one member in each of NAP, AtNAC3, TIP, and ATAF subfamilies also showed maturation-specific expression patterns. These findings provided important insights into the molecular mechanisms underlying loquat fruit quality formation.

### 3.8 NAC-mediated cultivar-specific sugar-acid networks in loquat flavor formation

**Figure 8:**
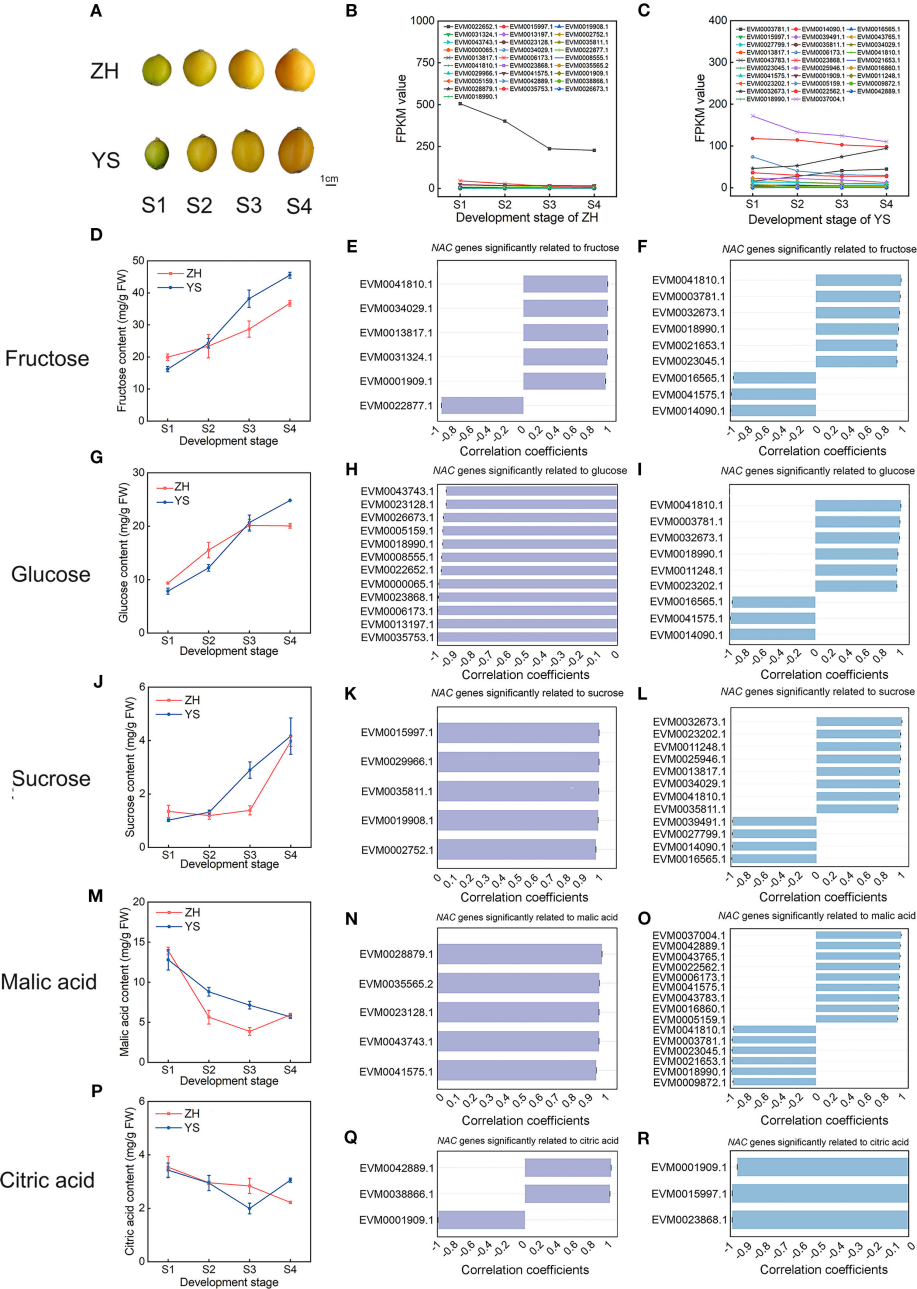
Analysis of sugar and acid content in various loquat cultivars and correlation with *NAC* gene expression. **(A)** Fruit photographs of two loquat cultivars at developmental stages S1-S4. **(B)** Expression patterns of *NAC* genes in ‘ZH’ loquat fruit. **(C)** Expression patterns of *NAC* genes in ‘YS’ loquat fruit. Changes in sugar and acid content are shown: **(D)** fructose, **(G)** glucose, **(J)** sucrose, **(M)** malic acid, **(P)** citric acid. Correlation analysis between NAC gene expression levels and fructose **(E)**, glucose **(H)**, sucrose **(K)**, malic acid **(N)**, and citric acid **(Q)** in ‘ZH’ loquat fruit. Correlation analysis between NAC gene expression levels and fructose **(F)**, glucose **(I)**, sucrose **(L)**, malic acid **(O)**, and citric acid **(R)** in ‘YS’ loquat fruit. The bar values represent the correlation coefficients (*R*-values).

This study systematically investigated the regulatory mechanisms of sugar-acid components on flavor quality in loquat fruits (**Figure 8**). Through combined analysis of sugar-acid content measurement and transcriptome association in two cultivars across four developmental stages (S1-S4), we found that: during fruit development, the contents of three soluble sugars (fructose, glucose and sucrose) continuously increased with a gradual rise in fructose proportion. Quantitative analysis revealed that, during the S4 stage, fructose levels exceeded those of glucose 1–2 folds and surpassed sucrose concentrations 9–11 folds. While malic acid content consistently decreased, the content of malic acid during S4 stage was lower 2–3 folds compared with S1 stage. Citric acid accumulation exhibited cultivar-specific patterns - showing a continuous decline in ‘ZH’ but a dynamic change of initial decrease (S1-S3) followed by increase (S3-S4) in ‘YS’. Gene expression analysis revealed 27 and 28 genes significantly correlated with sugar-acid contents in ‘YS’ and ‘ZH’ respectively. Notably, *EVM0032673.1* expression was significantly correlated with all three sugars. And the number of genes associated with malic acid was the largest (14) while those with citric acid were the smallest (3). Importantly, five core genes (*EVM0041810.1*, *EVM0018990.1*, *EVM0035811.1*, *EVM0041575.1* and *EVM0001909.1*) maintained significant correlations in both cultivars, whereas *EVM0022652.1* (specifically associated with glucose in ‘ZH’) and *EVM0037004.1* (specifically associated with malic acid in ‘YS’) displayed cultivar-specific regulatory characteristics (**Supplementary Figures 1**, **2**). Remarkably, these key regulatory genes maintained high expression levels throughout all fruit developmental stages, collectively forming a sophisticated regulatory network for sugar-acid metabolism in loquat. Particularly, *EVM0022652.1* demonstrated significantly higher expression abundance compared to other *NAC* family members, suggesting its potential critical role in fruit development or flavor quality formation in loquat. These findings provided important clues for deciphering the molecular mechanisms underlying loquat flavor quality formation.

## 4 Discussion

This study systematically elucidated the evolutionary characteristics and functional differentiation mechanisms of the NAC transcription factor family in loquat, providing novel insights into gene family expansion and functional specialization in Rosaceae plants. A total of 117 *EjNAC* genes were identified in the loquat (*Eriobotrya japonica*) genome. Notably, although 228 *NAC* genes have been reported in the apple (*Malus domestica*) genome (Geng et al., 2022), genome size-normalized comparative analysis revealed that the *NAC* gene density per genomic unit in loquat (2n=34) was significantly higher than that in apple (2n=17). The significant expansion of the ANAC001 and NAC2 subfamilies was particularly noteworthy, suggesting these subfamilies might play central roles in loquat-specific sugar-acid metabolism regulation.

Evolutionary selection pressure analysis revealed the dominant role of purifying selection in maintaining functional conservation of *NAC* genes (96.58% with *K*a/*K*s<1.0, **Supplementary Table 3**), similar to findings in *Dendrobium catenatum* NAC family studies (100%) (Liu et al., 2024c), indicating transcription factor families generally experienced strong functional constraints. However, the significantly elevated gene collinearity between loquat and closely related Rosaceae species (248 homologous pairs with pear, 231 with apple) reflected lineage-specific adaptations, supporting the recently proposed “conserved core-variable periphery” transcription factor evolution model (Mitra et al., 2011). The high-density clustering of ANAC001 subfamily members on chromosome LG16 (12/17) was particularly interesting, a non-random distribution pattern resembling functional synergy phenomena observed in bayberry *AP2/ERF* gene clusters (Liu et al., 2024b), suggesting Rosaceae plants might achieve precise metabolic pathway regulation through localized chromosomal gene enrichment.

*Cis*-regulatory element analysis demonstrated the predominance of light-responsive elements (44.2%) in loquat *NAC* genes, closely corresponding with photosynthate allocation demands during late fruit development. It is noteworthy that the simultaneous presence of ABA, MeJA and SA response elements in genes such as *EVM0032673.1* is particularly remarkable, indicating that these genes may coordinate sugar-acid balance by integrating multiple hormone signals—a regulatory process closely associated with fruit ripening.

Cross-cultivar comparisons revealed that the cultivar-specific gene *EVM0022652.1* in ‘ZH’ showed a strong negative correlation with glucose accumulation (r=-0.98, *P* < 0.05), explaining sugar content variations between cultivars. This finding aligned with cultivar-specific regulatory loci identified in recent GWAS studies on apple sugar-acid components (Lin et al., 2023).

NAC transcription factors are plant-specific regulators that play pivotal roles in growth, secondary metabolism, and stress responses across plant species (Liu et al., 2024d; Valoroso et al., 2022). Recent studies have systematically elucidated the pivotal role of NAC transcription factors in regulating fruit sugar-acid metabolism and flavor quality formation (Khan et al., 2025). In Rosaceae fruit trees such as apple and peach, specific NAC family members have been demonstrated to precisely modulate fruit flavor quality through fine-tuning sugar-acid metabolic networks. Taking peach (*Prunus persica*) as an example, research has shown that the NAC transcription factor PpNAP4 directly activated the expression of sucrose synthase gene (*PpSUS1*) and sucrose phosphate synthase gene (*PpSPS2*), significantly promoting sucrose accumulation during fruit ripening (Dai et al., 2025a). Additionally, the latest pan-genome analysis demonstrated that NAC transcription factor PpBL interacts with PpNAC1 to specifically activate the promoter activity of malate transporter *PpALMT4*, thereby enhancing vacuolar malate accumulation (Chen et al., 2025).

Our findings in loquat further extend this understanding: through integrated transcriptomic and metabolomic analyses across cultivars, we identified that the ‘ZH’-specific gene *EVM0022652.1* exhibited a significant negative correlation with glucose accumulation (r=-0.98, *P* <0.05). Intriguingly, this gene demonstrated extraordinary expression levels that were 200-500-fold higher than other *NAC* members throughout fruit development, while showing a progressive decrease during ripening - a pattern that was inversely correlated with glucose accumulation trends. Notably, such strong negative regulation of sugar accumulation by NAC transcription factors had rarely been reported in fruit crops, suggesting that *EVM0022652.1* might represent a novel regulatory factor involved in sugar metabolism in loquat. These findings not only provided valuable molecular markers for breeding programs but also revealed an unconventional transcriptional regulator that could be exploited for precisely modulating sugar composition in loquat fruits.

Based on these findings, functional validation of selected *NAC* gene family members can be conducted *in planta* through transient expression and transgenic approaches to elucidate their pivotal regulatory roles in sugar-acid metabolism. This should be integrated with systematic protein-protein interaction analyses to establish interaction networks between NAC transcription factors and key enzymes involved in sugar-acid metabolism (e.g., sucrose phosphate synthase, malate dehydrogenase), thereby elucidating the molecular mechanisms underlying NAC-mediated regulation of fruit quality attributes. Such a multi-tiered experimental validation system will facilitate comprehensive dissection of the molecular networks through which the loquat *NAC* gene family modulates fruit flavor quality, ultimately providing precise molecular targets for quality improvement.

## 5 Conclusion

This study represented the first systematic identification and functional characterization of the NAC transcription factor family at the whole-genome level in loquat. Through comprehensive bioinformatics analysis, we identified 117 *NAC* family members and classified them into 14 distinct subfamilies based on phylogenetic relationships. Chromosomal localization revealed an uneven distribution pattern of these genes across the 17 loquat chromosomes, with 3 additional members located in genomic scaffold regions. Collinearity analysis demonstrated that loquat *NAC* genes had primarily undergone purifying selection during evolution and exhibited high homology with *NAC* genes from other Rosaceae species, providing valuable insights into the evolution and functional diversification of *NAC* genes within the Rosaceae family. *Cis*-acting element prediction analysis suggested that loquat *NAC* genes were likely involved in various crucial biological processes, including abiotic stress responses, hormone signaling transduction, and growth regulation. Expression pattern analysis based on transcriptome data further uncovered differential expression profiles of *NAC* genes among different loquat cultivars and during fruit development. Notably, correlation analysis successfully identified several *NAC* candidate genes that were significantly associated with fruit sugar-acid content, which might play direct roles in regulating loquat fruit flavor quality.

Our findings not only filled a critical gap in loquat functional genomics research but also established a theoretical foundation for understanding the regulatory networks of NAC transcription factors in loquat growth, development, and fruit quality formation. Moreover, this study provided valuable candidate gene resources for subsequent molecular breeding and quality improvement research in loquat. Future studies would focus on functional validation of these candidate genes to elucidate the molecular mechanisms by which NAC transcription factors regulate loquat fruit flavor quality, thereby offering theoretical support and technical guidance for the high-quality development of the loquat industry.

## Data Availability

The datasets presented in this study can be found in online repositories. The names of the repository/repositories and accession number(s) can be found in the article/**Supplementary Material**.

## Supplementary material

Supplementary Figure 1 Heatmap analysis of correlation between *NAC* gene expression and sugar-acid content in ‘Yingshuang’ (YS) loquat.

Supplementary Figure 2 Heatmap analysis of correlation between *NAC* gene expression and sugar-acid content in ‘Zhehong NO.16’ (ZH) loquat.
