# Supplementary figures and images for: Identification and evolutionary analysis of NAC transcription factors in Eriobotrya japonica: implications for sugar-acid regulatory networks during fruit development
Source: Front Plant Sci. 2025 Sep 26;16:1671017. doi: 10.3389/fpls.2025.1671017 (PMC12511720; doi:10.3389/fpls.2025.1671017)

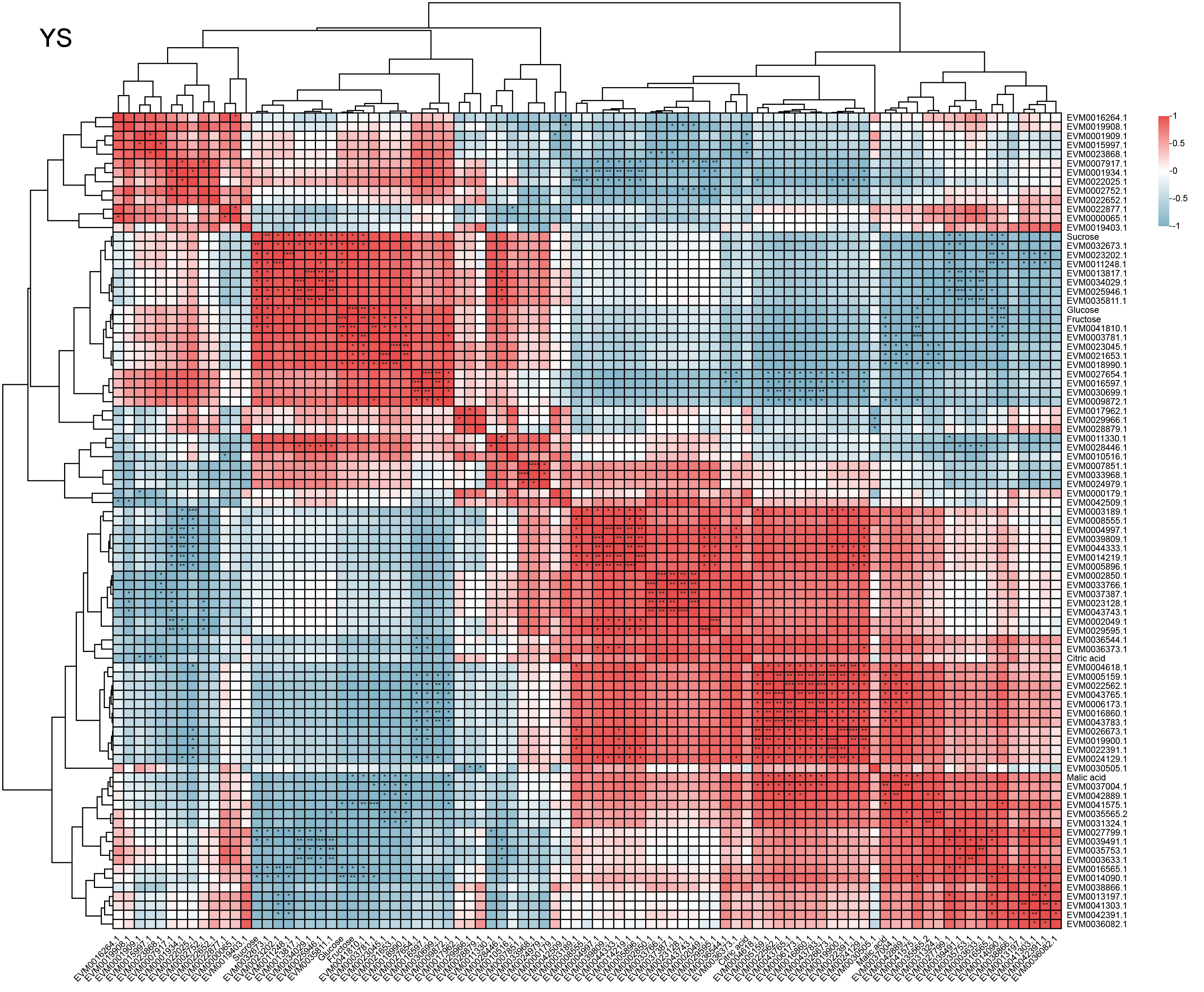

Supplement: Supplementary Figure 1 — Heatmap analysis of correlation between NAC gene expression and sugar-acid content in ‘Yingshuang’ (YS) loquat. [file Image1.tiff]

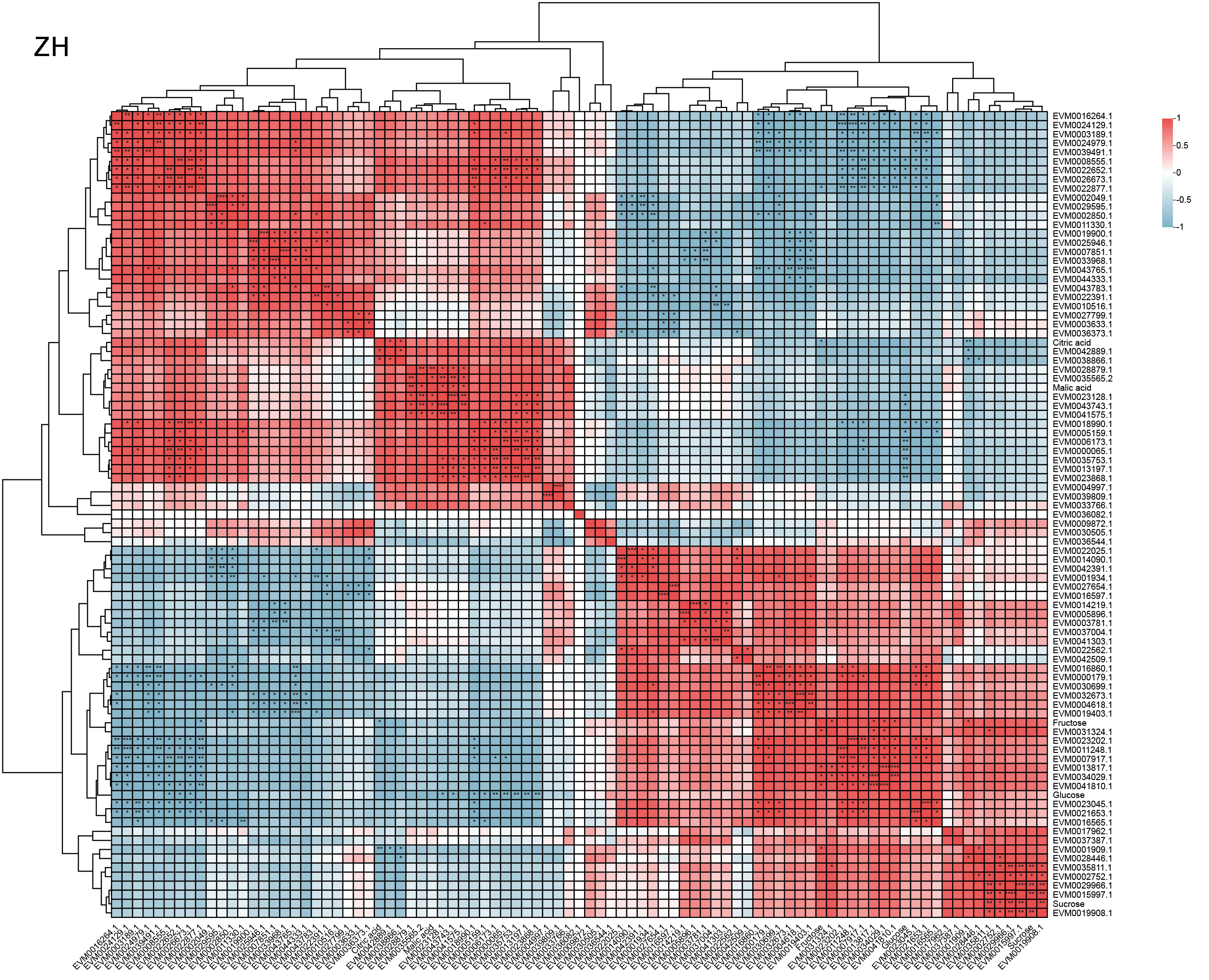

Supplement: Supplementary Figure 2 — Heatmap analysis of correlation between NAC gene expression and sugar-acid content in ‘Zhehong NO.16’ (ZH) loquat. [file Image2.tiff]
