# Supplementary material for: Identification and evolutionary analysis of NAC transcription factors in Eriobotrya japonica: implications for sugar-acid regulatory networks during fruit development
Source: Front Plant Sci. 2025 Sep 26;16:1671017. doi: 10.3389/fpls.2025.1671017 (PMC12511720; doi:10.3389/fpls.2025.1671017)
Supplement: Supplementary file 3 [file Table1.docx]

**Supplementary Table 1.** Basic information of *NAC* genes in loquat.

|  | ID | Molecular weight (Da) | Amino Acids | Isoelectric point | Subcellular Localization |
| --- | --- | --- | --- | --- | --- |
| OsNAC7 | EVM0021653.1 | 41885.54 | 369 | 7.13 | Nuclear |
|  | EVM0007598.1 | 45510.38 | 398 | 6.22 | Nuclear |
|  | EVM0023868.1 | 47411.6 | 416 | 6.31 | Nuclear |
|  | EVM0008045.1 | 47966.09 | 422 | 6.09 | Nuclear |
|  | EVM0019415.1 | 47279.25 | 417 | 5.97 | Nuclear |
|  | EVM0010978.1 | 45848.95 | 399 | 5.79 | Nuclear |
|  | EVM0022762.1 | 46175.26 | 401 | 5.86 | Nuclear |
|  | EVM0023045.1 | 41997.64 | 369 | 6.99 | Nuclear |
|  | EVM0000532.1 | 44805.63 | 395 | 6.89 | Nuclear |
|  | EVM0023658.1 | 44879.68 | 395 | 7.11 | Nuclear |
|  | EVM0045184.1 | 44859.62 | 395 | 6.76 | Nuclear |
|  | EVM0025946.1 | 39845.36 | 345 | 4.83 | Nuclear |
|  | EVM0042509.1 | 39681.22 | 345 | 4.93 | Nuclear |
|  | EVM0014483.1 | 36714.98 | 315 | 6.06 | Nuclear |
|  | EVM0033766.1 | 36034.05 | 311 | 5.91 | Nuclear |
|  | EVM0035565.2 | 37135.44 | 321 | 6.10 | Nuclear |
|  | EVM0038864.1 | 37088.47 | 321 | 6.23 | Nuclear |
|  | EVM0002850.1 | 43248.09 | 372 | 6.20 | Nuclear |
|  | EVM0029966.1 | 43113.84 | 370 | 6.49 | Nuclear |
| NAM | EVM0000179.1 | 39937.79 | 352 | 6.28 | Nuclear |
|  | EVM0037387.1 | 40277.22 | 353 | 6.31 | Nuclear |
|  | EVM0016860.1 | 40418.43 | 361 | 6.47 | Nuclear |
|  | EVM0041575.1 | 40562.72 | 361 | 7.68 | Nuclear |
|  | EVM0001909.1 | 53526.68 | 480 | 6.79 | Nuclear |
|  | EVM0007917.1 | 53303.33 | 478 | 6.86 | Nuclear |
|  | EVM0037272.1 | 41524.06 | 370 | 6.87 | Nuclear |
|  | EVM0022754.1 | 41257.06 | 368 | 8.51 | Nuclear |
|  | EVM0005183.1 | 38534.42 | 347 | 8.95 | Nuclear |
|  | EVM0040046.1 | 38534.42 | 347 | 8.95 | Nuclear |
|  | EVM0038241.1 | 37996.62 | 343 | 7.13 | Nuclear |
|  | EVM0024948.1 | 38274.96 | 345 | 7.61 | Nuclear |
|  | EVM0011248.1 | 46561.79 | 412 | 6.25 | Nuclear |
|  | EVM0023202.1 | 44348.63 | 393 | 6.27 | Nuclear |
| NAC1 | EVM0005159.1 | 31268.49 | 271 | 5.74 | Chloroplast |
|  | EVM0043783.1 | 31420.54 | 273 | 5.44 | Chloroplast |
| NAC2 | EVM0033968.1 | 65147.47 | 581 | 4.65 | Cytoplasm |
|  | EVM0007851.1 | 62251.09 | 558 | 4.61 | Cytoplasm |
|  | EVM0003189.1 | 67792.25 | 607 | 4.65 | Cytoplasm |
|  | EVM0009872.1 | 44184.4 | 395 | 4.93 | Cytoplasm |
|  | EVM0010516.1 | 43094.04 | 388 | 5.18 | Nuclear |
|  | EVM0036544.1 | 61859.93 | 557 | 4.67 | Nuclear |
|  | EVM0004618.1 | 64453.48 | 574 | 4.72 | E.R |
|  | EVM0036082.1 | 64453.75 | 575 | 4.80 | E.R |
| TIP | EVM0044333.1 | 60465.93 | 541 | 5.27 | Nuclear |
|  | EVM0032673.1 | 61952.61 | 556 | 4.89 | Nuclear |
|  | EVM0022562.1 | 94421.56 | 851 | 4.82 | Nuclear |
| OsNAC8 | EVM0042889.1 | 42811.9 | 382 | 6.73 | vacuole |
|  | EVM0038866.1 | 44516.74 | 398 | 6.54 | vacuole |
| ANAC011 | EVM0028879.1 | 42016.77 | 370 | 5.46 | Nuclear |
|  | EVM0016264.1 | 41980.87 | 369 | 5.12 | Nuclear |
|  | EVM0019900.1 | 45833.31 | 405 | 4.97 | Chloroplast |
|  | EVM0035753.1 | 39870.12 | 351 | 4.78 | Nuclear |
|  | EVM0005896.1 | 29753.9 | 259 | 5.14 | Nuclear |
|  | EVM0027174.1 | 29982.26 | 260 | 5.23 | Nuclear |
|  | EVM0026673.1 | 78935.44 | 693 | 5.61 | Nuclear |
|  | EVM0018990.1 | 79083.77 | 694 | 5.61 | Nuclear |
| NAP | EVM0015997.1 | 40321.31 | 354 | 6.99 | Nuclear |
|  | EVM0016565.1 | 40075.36 | 356 | 7.51 | Nuclear |
|  | EVM0007593.1 | 39204.58 | 347 | 9.27 | Nuclear |
|  | EVM0019403.1 | 32079.37 | 279 | 6.46 | Nuclear |
|  | EVM0042391.1 | 32223.45 | 279 | 8.29 | Nuclear |
|  | EVM0022652.1 | 41896.28 | 372 | 9.05 | Nuclear |
|  | EVM0041303.1 | 41987.29 | 377 | 8.83 | Nuclear |
|  | EVM0014090.1 | 40986.99 | 370 | 6.98 | Nuclear |
|  | EVM0022025.1 | 41090.97 | 369 | 7.80 | Nuclear |
|  | EVM0003781.1 | 40949 | 367 | 7.23 | Nuclear |
| ATAF | EVM0037004.1 | 35608.24 | 308 | 6.26 | Nuclear |
|  | EVM0017962.1 | 35247.91 | 305 | 7.72 | Nuclear |
|  | EVM0014219.1 | 33990.19 | 296 | 7.59 | Nuclear |
|  | EVM0030699.1 | 33014.31 | 288 | 8.03 | Nuclear |
| AtNAC3 | EVM0027546.1 | 46258.73 | 407 | 6.28 | Nuclear |
|  | EVM0022861.1 | 51333.11 | 455 | 6.49 | Nuclear |
|  | EVM0033669.1 | 38539.09 | 338 | 6.86 | Nuclear |
|  | EVM0001934.1 | 37886.57 | 337 | 8.71 | Nuclear |
|  | EVM0016597.1 | 36065.39 | 326 | 8.13 | Nuclear |
|  | EVM0027654.1 | 37781.54 | 338 | 8.76 | Nuclear |
| ONAC022 | EVM0031324.1 | 35222.51 | 305 | 7.61 | Nuclear |
|  | EVM0045591.1 | 34573.88 | 300 | 8.54 | Nuclear |
|  | EVM0002752.1 | 35431.74 | 309 | 6.05 | Nuclear |
|  | EVM0019774.1 | 35107.68 | 306 | 8.05 | Chloroplast |
|  | EVM0019908.1 | 37736.93 | 326 | 6.70 | Nuclear |
|  | EVM0027473.1 | 37704.77 | 326 | 6.35 | Nuclear |
|  | EVM0032555.1 | 38778.93 | 335 | 6.35 | Nuclear |
|  | EVM0034269.1 | 42377.56 | 377 | 6.84 | Nuclear |
|  | EVM0029950.1 | 44180.58 | 392 | 8.57 | Cytoplasm |
|  | EVM0001227.1 | 51895.85 | 466 | 6.68 | Cytoplasm |
|  | EVM0044081.1 | 52503.63 | 471 | 8.28 | Cytoplasm |
| TERN | EVM0004184.1 | 54366.06 | 479 | 6.57 | Nuclear |
|  | EVM0022137.1 | 54310.89 | 476 | 6.56 | Nuclear |
|  | EVM0043765.1 | 35943.12 | 314 | 7.74 | Nuclear |
|  | EVM0013197.1 | 33751.56 | 293 | 7.76 | Nuclear |
|  | EVM0035599.1 | 32525.02 | 286 | 6.07 | Nuclear |
|  | EVM0022936.1 | 32293.9 | 282 | 7.07 | Nuclear |
|  | EVM0030505.1 | 31550.13 | 281 | 6.33 | Nuclear |
|  | EVM0000065.1 | 31550.13 | 281 | 6.33 | Nuclear |
|  | EVM0023128.1 | 28963.19 | 254 | 5.79 | Nuclear |
|  | EVM0043743.1 | 28334.43 | 248 | 5.60 | Nuclear |
| ANAC001 | EVM0029595.1 | 26733.15 | 233 | 6.25 | Nuclear |
|  | EVM0002049.1 | 25085.17 | 222 | 5.90 | Nuclear |
|  | EVM0039809.1 | 50478.65 | 446 | 4.54 | Nuclear |
|  | EVM0004997.1 | 56163.9 | 500 | 4.92 | Nuclear |
|  | EVM0034029.1 | 37475.85 | 335 | 5.21 | Nuclear |
|  | EVM0013817.1 | 45436.97 | 399 | 4.90 | Nuclear |
|  | EVM0041810.1 | 43532.42 | 386 | 4.76 | Nuclear |
|  | EVM0036373.1 | 51107.75 | 456 | 4.94 | Nuclear |
|  | EVM0035811.1 | 39646.95 | 349 | 5.10 | Nuclear |
|  | EVM0028446.1 | 36463.64 | 319 | 4.96 | Nuclear |
|  | EVM0011330.1 | 47513.25 | 412 | 4.93 | Nuclear |
|  | EVM0022877.1 | 50646.84 | 442 | 9.21 | Nuclear |
|  | EVM0024129.1 | 64158.1 | 567 | 5.42 | vacuole |
|  | EVM0024979.1 | 59401.3 | 525 | 6.10 | Chloroplast |
| SENU5 | EVM0039491.1 | 28733.57 | 255 | 8.81 | Nuclear |
|  | EVM0022391.1 | 28862.79 | 255 | 8.97 | Chloroplast |
|  | EVM0003633.1 | 22920.1 | 201 | 9.31 | Nuclear |
|  | EVM0027799.1 | 25591.05 | 226 | 9.14 | Nuclear |
|  | EVM0006173.1 | 30307.25 | 263 | 9.35 | Nuclear |
|  | EVM0008555.1 | 30369.29 | 263 | 9.27 | Nuclear |
