# Supplementary material for: Identification and evolutionary analysis of NAC transcription factors in Eriobotrya japonica: implications for sugar-acid regulatory networks during fruit development
Source: Front Plant Sci. 2025 Sep 26;16:1671017. doi: 10.3389/fpls.2025.1671017 (PMC12511720; doi:10.3389/fpls.2025.1671017)
Supplement: Supplementary file 4 [file Table2.docx]

**Supplementary Table 2**. Distribution of plant *NAC* genes across phylogenetic groups.

| **Species**  **Groups** | ***Arabidopsis thaliana*** | ***Malus domestica*** | ***Cerasus humilis*** | ***Eriobotrya japonica*** | ***Actinidia chinensis*** | ***Vitis vinifera* L*.*** |
| --- | --- | --- | --- | --- | --- | --- |
| ANAC011 | 8 | 13 | 2 | 8 | 11 | 4 |
| NAM | 11 | 16 | 2 | 14 | 17 | 7 |
| NAC1 | 2 | 5 | 1 | 2 | 4 | 2 |
| OsNAC7 | 13 | 23 | 7 | 19 | 14 | 8 |
| OsNAC8 | 3 | 2 | 2 | 2 | 0 | 1 |
| TIP | 4 | 3 | 8 | 3 | 2 | 8 |
| NAC2 | 9 | 8 | 7 | 8 | 3 | 4 |
| ONAC022 | 5 | 8 | 6 | 11 | 14 | 11 |
| TERN | 2 | 11 | 2 | 10 | 5 | 3 |
| ANAC001 | 6 | 35 | 15 | 14 | 2 | 0 |
| ONAC003 | 4 | 10 | 11 | 0 | 9 | 2 |
| SENU5 | 2 | 4 | 2 | 6 | 10 | 3 |
| ANAC063 | 13 | 9 | 3 | 0 | 10 | 0 |
| AtNAC3 | 3 | 0 | 1 | 6 | 0 | 1 |
| ATAF | 4 | 1 | 2 | 4 | 4 | 3 |
| NAP | 5 | 15 | 5 | 10 | 14 | 8 |
| Unclassified | 0 | 65 | 0 | 0 | 37 | 9 |
| Total | 94 | 228 | 76 | 117 | 156 | 74 |
