# Supplementary material for: Identification and evolutionary analysis of NAC transcription factors in Eriobotrya japonica: implications for sugar-acid regulatory networks during fruit development
Source: Front Plant Sci. 2025 Sep 26;16:1671017. doi: 10.3389/fpls.2025.1671017 (PMC12511720; doi:10.3389/fpls.2025.1671017)
Supplement: Supplementary file 5 [file Table3.docx]

**Supplementary Table 3.** The *K*a/*K*s of *NAC* genes in loquat.

| Seq_1 | Seq_2 | *K*a | *K*s | *K*a/*K*s |
| --- | --- | --- | --- | --- |
| EVM0041806.1 | EVM0014090.1 | 0.15486359738763558 | 0.26206381734067896 | 0.5909384933758912 |
| EVM0045591.1 | EVM0031324.1 | 0.0678595243670979 | 0.24557859281527963 | 0.27632508024891556 |
| EVM0021653.1 | EVM0023045.1 | 0.02659591185936527 | 0.14736494170468256 | 0.18047652007126047 |
| EVM0019900.1 | EVM0035753.1 | 0.03521552224636615 | 0.0981718656345365 | 0.35871297768204435 |
| EVM0032555.1 | EVM0019908.1 | 0.044268547456795086 | 0.15480256758878513 | 0.28596778558860403 |
| EVM0043783.1 | EVM0005159.1 | 0.03562942220436799 | 0.1597032194292297 | 0.223097707934164 |
| EVM0013197.1 | EVM0043765.1 | 0.04249055659029395 | 0.21609216928798458 | 0.1966316351504023 |
| EVM0014483.1 | EVM0033766.1 | 0.04124029711498793 | 0.11151722149353091 | 0.369811017192356 |
| EVM0036082.1 | EVM0004618.1 | 0.04543020203232619 | 0.13109670865549497 | 0.34653960803631484 |
| EVM0009872.1 | EVM0010516.1 | 0.0727008521511299 | 0.1726624494872025 | 0.4210576901176094 |
| EVM0045184.1 | EVM0000532.1 | 0.0392390798494314 | 0.21795846396500645 | 0.18003008066587997 |
| EVM0041806.1 | EVM0022025.1 | 0.30714030990371427 | 1.7813336527967223 | 0.17242155023653152 |
| EVM0041806.1 | EVM0003781.1 | 0.29537966047987396 | 2.171149610612985 | 0.13604758466943181 |
| EVM0014090.1 | EVM0001934.1 | 0.4812084861869248 | NaN | NaN |
| EVM0014090.1 | EVM0003781.1 | 0.22747145462205445 | 2.1373005841232358 | 0.10642932319010612 |
| EVM0022936.1 | EVM0023128.1 | 0.41522931571804667 | 2.313714300595191 | 0.17946438573303156 |
| EVM0025946.1 | EVM0042509.1 | 0.024911475321010533 | 0.14249901837139076 | 0.1748185749328078 |
| EVM0014219.1 | EVM0037004.1 | 0.27357464856417935 | 1.7695717439779228 | 0.1545993540500342 |
| EVM0022936.1 | EVM0043743.1 | 0.38648921880000314 | 1.6389631027232208 | 0.2358132517796353 |
| EVM0016860.1 | EVM0037387.1 | 0.2817068114068708 | 1.6692522066800457 | 0.16876228186461642 |
| EVM0016860.1 | EVM0000179.1 | 0.29267551539305414 | 1.5980415017320944 | 0.18314637953759483 |
| EVM0016860.1 | EVM0041575.1 | 0.05228061240280488 | 0.21154491767974443 | 0.2471371705651276 |
| EVM0022754.1 | EVM0037272.1 | 0.03869906003543142 | 0.12792115619798075 | 0.30252275061943423 |
| EVM0004184.1 | EVM0022137.1 | 0.050878933589648484 | 0.14968961892541305 | 0.33989620626264205 |
| EVM0014219.1 | EVM0017962.1 | 0.26288885932862704 | 2.133917701652813 | 0.12319540679802603 |
| EVM0001653.1 | EVM0000065.1 | 0.12302229300923717 | 0.23568682487737724 | 0.5219735684132663 |
| EVM0007917.1 | EVM0001909.1 | 0.05946465572111771 | 0.11663662133000419 | 0.5098283458749394 |
| EVM0022652.1 | EVM0041303.1 | 0.04340422420107861 | 0.12338769912033559 | 0.3517710801848086 |
| EVM0007593.1 | EVM0016565.1 | 0.057676872048577736 | 0.17577116099517331 | 0.32813614999198615 |
| EVM0042889.1 | EVM0038866.1 | 0.057686787288205255 | 0.11747616360714015 | 0.4910509972143751 |
| EVM0023128.1 | EVM0043743.1 | 0.06774960041115241 | 0.12115354233163711 | 0.5592044533514295 |
| EVM0038241.1 | EVM0024948.1 | 0.040710211415120226 | 0.32464806172220856 | 0.1253979808139274 |
| EVM0001934.1 | EVM0003781.1 | 0.508085735280444 | 2.3555194395364003 | 0.21570008158389153 |
| EVM0023128.1 | EVM0000065.1 | 0.43371962333370634 | 2.442476179204005 | 0.17757373726979567 |
| EVM0003189.1 | EVM0000395.1 | 0.638201400437689 | 1.7421313896785724 | 0.36633367851516574 |
| EVM0003633.1 | EVM0039491.1 | 0.23912600174413104 | 1.3984763665016364 | 0.17099037743649365 |
| EVM0003633.1 | EVM0022391.1 | 0.23760575799679096 | 1.5691238478561675 | 0.151425751588329 |
| EVM0022762.1 | EVM0008045.1 | 0.31820670584965427 | NaN | NaN |
| EVM0003633.1 | EVM0006173.1 | 0.4975347786367541 | 1.6479184330021686 | 0.3019171147508485 |
| EVM0003633.1 | EVM0008555.1 | 0.4934392189127538 | 2.066575668670997 | 0.23877142579060834 |
| EVM0022762.1 | EVM0010978.1 | 0.03223072176885207 | 0.1373690454052633 | 0.2346287089188519 |
| EVM0003633.1 | EVM0027799.1 | 0.058973833450474425 | 0.24630131996142743 | 0.23943774828210485 |
| EVM0027174.1 | EVM0005896.1 | 0.015067471042759985 | 0.15274565462171236 | 0.09864418781716483 |
| EVM0040869.1 | EVM0022562.1 | 0.10010004315883961 | 0.18923711896952566 | 0.528966218170757 |
| EVM0016425.1 | EVM0005183.1 | 0.026281189500135367 | 0.1768681537556976 | 0.1485919818919846 |
| EVM0040869.1 | EVM0032673.1 | 0.6086062701810704 | 2.173561422241595 | 0.2800041737736652 |
| EVM0022762.1 | EVM0007598.1 | 0.3265455876639442 | NaN | NaN |
| EVM0037004.1 | EVM0017962.1 | 0.0719201312198464 | 0.2715860004259123 | 0.26481531119814095 |
| EVM0033669.1 | EVM0007276.1 | 0.13960549632893612 | 0.20923885927764868 | 0.667206353594612 |
| EVM0022861.1 | EVM0018940.1 | 0.13212407812065646 | 0.21277523985227476 | 0.6209560765265139 |
| EVM0039491.1 | EVM0022391.1 | 0.0275141901179971 | 0.12575117063590863 | 0.21879867979646736 |
| EVM0044081.1 | EVM0001227.1 | 0.038785438415394016 | 0.18755674088127722 | 0.20679309222986056 |
| EVM0019403.1 | EVM0042391.1 | 0.06801031810172589 | 0.26168470395243204 | 0.2598941286002277 |
| EVM0044081.1 | EVM0034269.1 | 0.2839233934897158 | 1.5960705518043654 | 0.17788899943598296 |
| EVM0039491.1 | EVM0006173.1 | 0.4782709648345588 | 1.6114370775854026 | 0.2967977909203914 |
| EVM0039491.1 | EVM0008555.1 | 0.45914038641598653 | 2.37641630864252 | 0.19320705077901998 |
| EVM0044081.1 | EVM0029950.1 | 0.324235452157685 | 1.5559763876817474 | 0.20838070212669751 |
| EVM0016264.1 | EVM0028879.1 | 0.058711149179941385 | 0.1892057291177489 | 0.31030323158662665 |
| EVM0023202.1 | EVM0011248.1 | 0.0794074071107872 | 0.2363759313025507 | 0.33593694025111737 |
| EVM0039491.1 | EVM0027799.1 | 0.2536694326202388 | 1.5252894692563057 | 0.16630904345253356 |
| EVM0001227.1 | EVM0034269.1 | 0.2652906209575498 | 1.948050693377172 | 0.13618260646884794 |
| EVM0022391.1 | EVM0006173.1 | 0.4564423443989116 | 1.9761350978091814 | 0.23097729750609713 |
| EVM0022391.1 | EVM0008555.1 | 0.5054314861457538 | 2.1406421003265184 | 0.23611209275415956 |
| EVM0001227.1 | EVM0029950.1 | 0.31834444484537905 | 1.620779793847725 | 0.1964143716831702 |
| EVM0022391.1 | EVM0027799.1 | 0.256577414763034 | 1.5831754705009105 | 0.16206505188073303 |
| EVM0016565.1 | EVM0015997.1 | 0.46146888577788925 | 2.030480857928009 | 0.22727073933058015 |
| EVM0043743.1 | EVM0000065.1 | 0.4189147117916329 | 1.590611331145659 | 0.2633671114928523 |
| EVM0035565.2 | EVM0002850.1 | 0.24944118154007963 | 1.2716246035505683 | 0.196159448978576 |
| EVM0044333.1 | EVM0022562.1 | 0.5030199108373599 | 2.4753023129879397 | 0.20321554591453683 |
| EVM0008045.1 | EVM0010978.1 | 0.3122116343535101 | 2.557122138357585 | 0.12209492447397943 |
| EVM0035811.1 | EVM0022562.1 | 0.6026624078551739 | 1.4721675022051863 | 0.40937081341113357 |
| EVM0008045.1 | EVM0007598.1 | 0.041001281480246926 | 0.2395249172819198 | 0.17117752067518133 |
| EVM0045361.1 | EVM0029595.1 | 0.47308741346585814 | 0.9138871619202229 | 0.5176650172783103 |
| EVM0024979.1 | EVM0024129.1 | 0.20799053779697613 | 0.23759624952041108 | 0.8753948693079365 |
| EVM0035565.2 | EVM0038864.1 | 0.020092729733106957 | 0.10933135567475732 | 0.18377829131543472 |
| EVM0044333.1 | EVM0032673.1 | 0.08476924548005363 | 0.15912468317246922 | 0.532722163463323 |
| EVM0006173.1 | EVM0008555.1 | 0.06350136225225267 | 0.18533967316007674 | 0.34262152926862527 |
| EVM0018990.1 | EVM0026673.1 | 0.038198681312386816 | 0.1476169856135665 | 0.2587688750966896 |
| EVM0034269.1 | EVM0029950.1 | 0.04664413296132933 | 0.24208004419728768 | 0.19268061981728582 |
| EVM0037387.1 | EVM0000179.1 | 0.06030874175461852 | 0.20018504662939993 | 0.3012649684382637 |
| EVM0002752.1 | EVM0019774.1 | 0.05261470137626634 | 0.15804835686599414 | 0.33290255222885506 |
| EVM0037387.1 | EVM0041575.1 | 0.23989217800165827 | 1.5871340788323929 | 0.15114802284261944 |
| EVM0006173.1 | EVM0027799.1 | 0.6350404792280199 | 1.798977834446251 | 0.3530007246717932 |
| EVM0002850.1 | EVM0029966.1 | 0.07477335365190754 | 0.1677438301465525 | 0.44575918879746823 |
| EVM0002850.1 | EVM0038864.1 | 0.24779926685274653 | 1.4526442442801084 | 0.17058496450763774 |
| EVM0008555.1 | EVM0027799.1 | 0.6498076354971775 | 2.121572961380013 | 0.30628578291952735 |
| EVM0000179.1 | EVM0041575.1 | 0.2677128945808538 | 1.561900410999371 | 0.17140202582414302 |
| EVM0010978.1 | EVM0007598.1 | 0.32398937681676776 | 2.9871541630233844 | 0.10846088254408966 |
| EVM0022562.1 | EVM0024129.1 | 0.7125141089442798 | 1.396671192541169 | 0.5101516468223979 |
| EVM0022562.1 | EVM0032673.1 | 0.5193372910854506 | NaN | NaN |
